# Evaluation of a range of mammalian and mosquito cell lines for use in Chikungunya virus research

**DOI:** 10.1038/s41598-017-15269-w

**Published:** 2017-11-07

**Authors:** Grace C. Roberts, Carsten Zothner, Roland Remenyi, Andres Merits, Nicola J. Stonehouse, Mark Harris

**Affiliations:** 1 School of Molecular and Cellular Biology, Faculty of Biological Sciences and Astbury Centre for Structural Molecular Biology, University of Leeds, Leeds, LS2 9JT UK; 2 Institute of Technology, University of Tartu, Tartu, Estonia

## Abstract

Chikungunya virus (CHIKV) is becoming an increasing global health issue which has spread across the globe and as far north as southern Europe. There is currently no vaccine or anti-viral treatment available. Although there has been a recent increase in CHIKV research, many of these *in vitro* studies have used a wide range of cell lines which are not physiologically relevant to CHIKV infection *in vivo*. In this study, we aimed to evaluate a panel of cell lines to identify a subset that would be both representative of the infectious cycle of CHIKV *in vivo*, and amenable to *in vitro* applications such as transfection, luciferase assays, immunofluorescence, western blotting and virus infection. Based on these parameters we selected four mammalian and two mosquito cell lines, and further characterised these as potential tools in CHIKV research.

## Introduction

Chikungunya virus (CHIKV) is an enveloped, positive sense, single stranded RNA virus^1^. CHIKV is in the alphavirus genus of the *Togaviridae* family and is transmitted by mosquitoes, mainly *Aedes aegypti* and *Aedes albopictus* ^2^. Prior to 2004, the virus was confined to Africa and Asia due to the distribution of *Aedes aegypti* populations^3^. However, recently mutations in the E1 and E2 glycoproteins (E1-A226V and E2-I211T respectively) have enabled one genotype of the virus (ECSA: East/Central/South African) to more efficiently infect *Aedes albopictus* due to enhanced infectivity of the mid-gut^4^. This mosquito can tolerate more moderate climates^5^. This, in addition to climate change and the expansion of international travel, has resulted in the global spread of CHIKV, causing large, disruptive outbreaks in naïve populations^6^. Recent outbreaks include La Reunion island^7^ and the Caribbean^8^, and it is now present across the Americas^9^ (though the Caribbean and American outbreaks are due to the Asian genotype, not the adapted ECSA) and Southern Europe, including France^10^ and Italy^11^. The *Aedes albopictus* mosquito is now established in many southern European countries including Spain, France, Italy, and Russia^12,13^. With increasing temperatures, it is likely that CHIKV will spread even further making it a concern for global health^14^.

Infection with CHIKV results in Chikungunya fever where patients develop a high fever, rash, and debilitating joint pain^15^. Although CHIKV infection is rarely fatal, the symptoms, particularly the joint pain, can persist for months. It has been reported that CHIKV causes significant inflammatory tissue damage^16^ and patients who have suffered from CHIKV are considerably more likely to develop arthritis^17^. In young children and the immunocompromised, serious neurological complications can occur, which can be fatal^18^. CHIKV has also been associated with altered cognitive abilities in infected children^19^.

CHIKV is introduced to the body via a mosquito bite. The virus initially infects and replicates in the local dermal fibroblasts, then disseminates through the body via the lymphatic and circulatory systems which it reaches target tissues; the liver, muscles, joints and (in some cases) brain^20,21^. There is some evidence that CHIKV infects non-human primates, which may act as a reservoir for the virus. However, animal intermediates are not required for spread of disease during outbreaks^22^. Despite being discovered in 1952^23^, little research was conducted on CHIKV before the last decade. *In vitro* CHIKV is promiscuous and infects a wide range of cell lines including Vero, HeLa and BHK cells^24–27^, many of which are of limited relevance to the *in vivo* situation. To address this we have evaluated and compared cell lines from a range of origins for their use with CHIKV – using both sub-genomic replicon (SGR) systems and infectious virus.

We propose that four mammalian cell lines, Huh7 (hepatocyte, human), C2C12 (myoblast, mouse), SVG-A (astroglia, human) and dermal fibroblasts (human, an in-house transformed cell line, see methods section for details) and two mosquito cell lines, U4.4 and C6/36 (both embryonic *Aedes albopictus*) provide good models for *in vitro* CHIKV research. They are all biologically and clinically relevant, and tractable for *in vitro* applications to investigate the molecular and cellular biology of CHIKV infection.

## Results

### Evaluation of mammalian cell lines for replication of the CHIKV SGR

We initially evaluated a panel of mammalian cell lines for their ability to support CHIKV genome replication. Cell lines were selected based on their relevance to CHIKV infection *in vivo* and their previous use in CHIKV research (e.g. HeLa and Vero cells). The origins of each cell line are shown in Table 1.

**Table 1:** Cell lines used, their origin and cell type.

Cell line	Species	Cell Type
Huh7^45^	Human	Liver, hepatocellular carcinoma (HCC)
C2C12^46^	Mouse	Muscle, myoblast
SVG-A^47^	Human	Brain, astroglia
Dermal fibroblasts^42^ (see methods)	Human	Dermal, fibroblast
HepG2^48^	Human	Liver, HCC
RD^49^	Human	Muscle, rhabdomyosarcoma
BHK-21^28^	Hamster	Kidney, fibroblast
A549^50^	Human	Lung, epithelial carcinoma
HeLa^51^	Human	Cervical, epithelial carcinoma
Vero E6^52^	African green monkey	Kidney, epithelial
A20^53^	Aedes aegypti	Embryonic
Aag2^54^	Aedes aegypti	Embryonic
U4.4^54^	Aedes albopictus	Embryonic
C6/36^54^	Aedes albopictus	Embryonic

**Figure 1:**
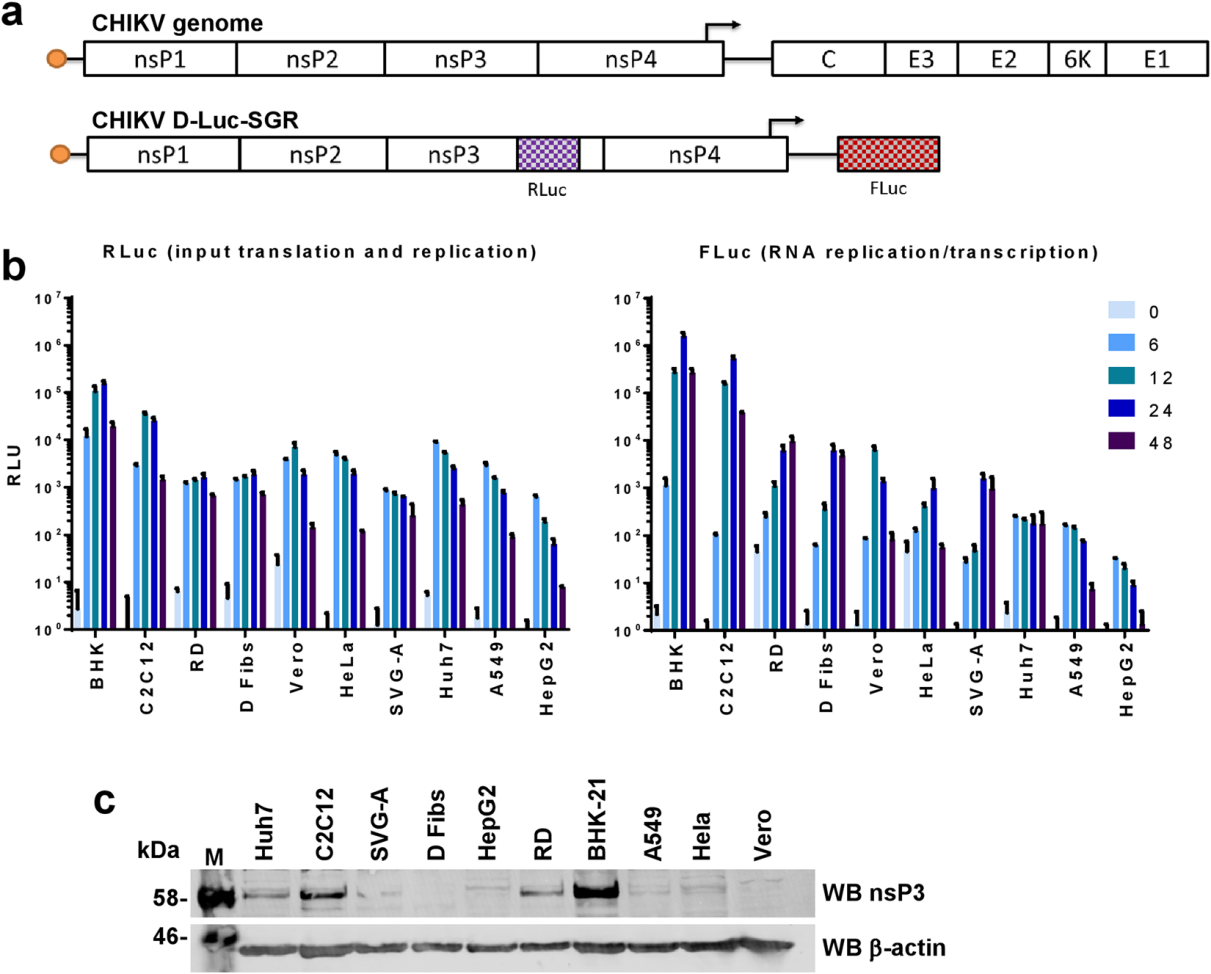
Mammalian cell lines transfected with CHIKV-D-Luc-SGR. (**a**) Schematic of the CHIKV genome and the corresponding dual luciferase replicon (CHIKV-D-luc-SGR). Renilla luciferase (Rluc) is present in the hypervariable region of nsP3, indicating both input translation and early replication levels. Firefly luciferase (Fluc) replaces the structural genes and is only expressed from a subgenomic RNA, thus indicating that RNA replication has occurred. Note that other SGRs used in this study have either no insertion into nsP3 (termed nsP3/SG-FLuc), or insertion of mCherry in place of Rluc and Gaussia luciferase in place of Fluc (nsP3-mCherry/SG-GLuc). **(b)** Liver (Huh7, HepG2), muscle (C2C12, RD), brain (SVG-A), fibroblasts (Dermal fibs, BHKs), as well as Vero, HeLa and A549 cells were transfected with CHIKV-D-Luc-SGR RNA. Cell lysates were taken at indicated time points over a 48 h period and luciferase assayed (n = 3 experimental replicates, all luciferase signal normalised to a mock-transfected control). **(c)** Western blot of nsP3 in mammalian cell lines. Cells were transfected with wildtype (untagged) nsP3/SG-FLuc replicon, lysed at 24 hpt and Western blotted for CHIKV nsP3.

To analyse CHIKV genome replication in these cell lines we exploited a CHIKV SGR (CHIKV-D-Luc-SGR) (Fig. 1a), derived from the ECSA strain (ICRES^28^). This construct contains two luciferase reporters, a *Renilla* luciferase gene (Rluc) is fused in frame within the C-terminal hypervariable domain of nsP3 and a firefly luciferase gene (Fluc) replaces the region encoding for structural proteins. The structural proteins are expressed from a subgenomic RNA transcribed from a promoter, located primarily within the coding region for the C-terminus of nsP4 but extending into the intragenic junction region, using the negative strand as a template. Thus Fluc expression is dependent on genome replication and transcription, whereas Rluc expression reflects both input RNA translation and early genome replication.

Cells were transfected with CHIKV-D-Luc-SGR RNA using lipofectamine (Fig. 1b). By 6 hpt, similarly high levels of RLuc were detected for all cell lines indicating that all had been successfully transfected to similar transfection efficiency. By 24 hpt, Rluc activities increased up to 10 fold for some cell lines (BHK-21, C2C12), remained at a stable level (RD) or slowly declined (A549). By 48 hpt Rluc values declined across the board, most likely resulting from cytotoxicity. In contrast, analysis of the FLuc levels over the 48 h time period revealed differences in the abilities of cells to support CHIKV RNA replication and transcription. BHK-21 cells produced the highest Fluc levels. Of the physiologically relevant cell lines, replication/transcription levels were highest in the muscle cell lines C2C12 and RD, followed by the dermal fibroblasts (an in-house transformed primary cell line, see methods section for more detail). The SVG-A astroglial cells supported moderate levels of replication, similar to Hela or Vero E6 cells. The two liver cell lines produced very different levels of replication, with Huh7 cells exhibiting moderate levels of replication whereas HepG2 cells appeared incapable of supporting replication. A549 cells facilitated low levels of replication. Vero E6 and HeLa cells, despite supporting moderate levels of replication at earlier time points, exhibited a drastic reduction in signal for replication by the 48 h time point.

When transfected with an SGR expressing native (untagged) nsP3 (CHIKV-FLuc-SGR), most of these cell lines expressed nsP3 to levels detectable by western blot (Fig. 1c). Predictably, those with the highest levels of replicon replication produced the strongest nsP3 signal with BHK-21, C2C12, and RD cells exhibiting the most intense bands. Most cell lines produced detectable nsP3 at varying intensities, though nsP3 was virtually undetectable in the dermal fibroblast and Vero E6 cells.

**Figure 2:**
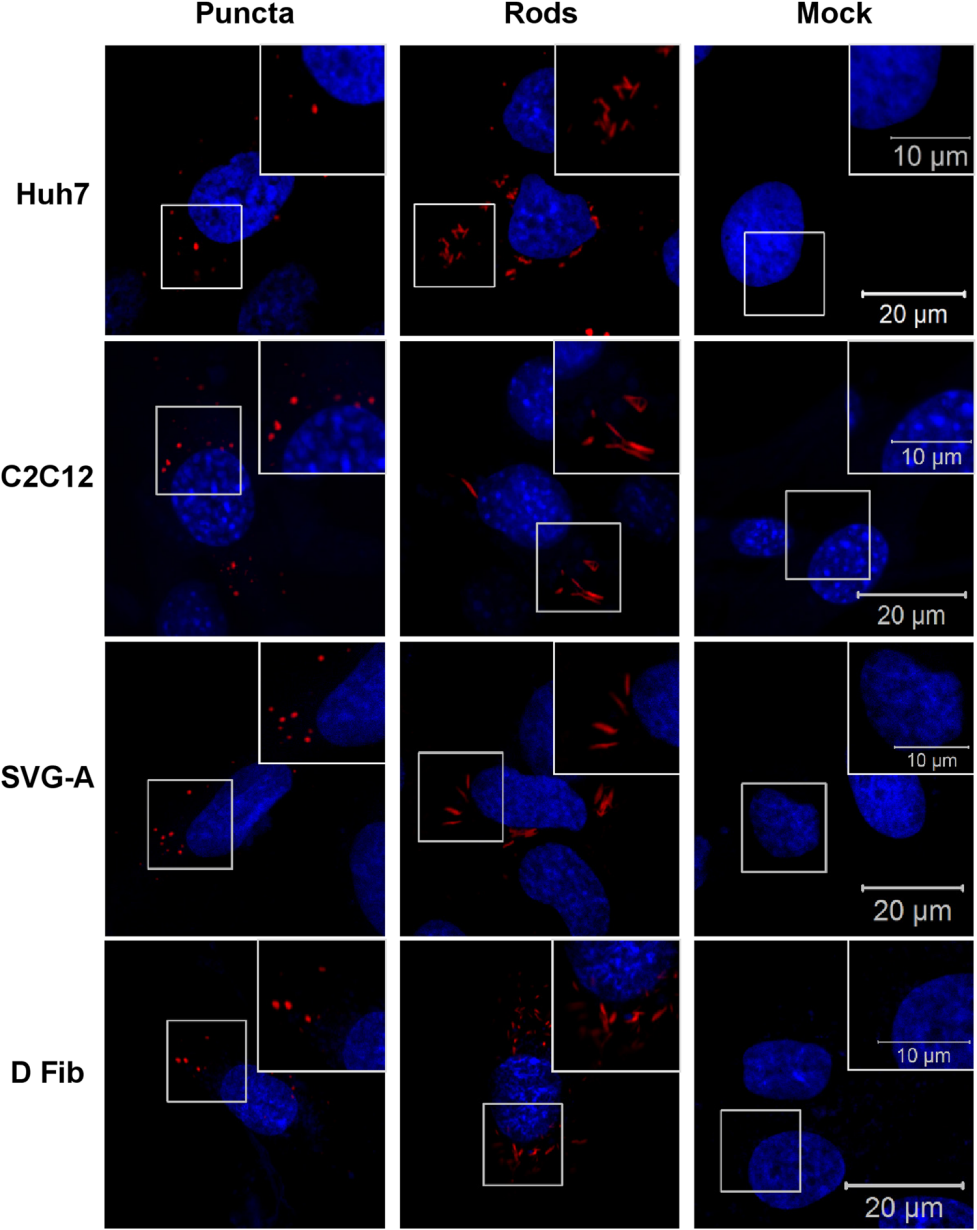
Cytoplasmic nsP3 organisation in mammalian cells. The indicated cell lines were transfected with nsP3-mCherry/SG-GLuc (expressing mCherry-tagged nsP3), two different nsP3 structures were observed; puncta and rods. The data for all 10 cell lines are shown in Supplementary Figure S1.

Previously our group has described different nsP3 structures in Huh7 cells^29^. Two different structures were observed in these cells – puncta and rods of nsP3, though the significance of these structures is currently unclear. To visualise nsP3 structures here, the cell lines were transfected with a mCherry-tagged nsP3 SGR, and fixed at 24 hpt. Images for the physiologically relevant cell types; Huh7, C2C12, SVG-A, and dermal fibroblasts are shown in Fig. 2 (data for all ten cell lines are shown in Supplementary Figure S1). All four cell types exhibited both puncta and rods. For the majority of cells for all lines, individual cells contained only one form of nsP3 organisation. IF data for all cell lines is shown in supplementary data Figure S1. Two of these cell lines, RD and HeLa cells, only formed puncta of nsP3 with no rods observed. Cells with low levels of subgenomic RNA synthesis varied greatly in appearance. In HepG2 cells, which grow in large clusters, the few nsP3-positive cells were always at the edges of the clusters. A549 cells had the smallest puncta of all the cell lines, with many puncta per cell. Although nsP3-positive cells were found in both HeLa and Vero E6 cells, the majority of these cells appeared to be either dead or dying.

From this data, we chose Huh7 (liver), C2C12 (myoblast), SVG-A (astroglia) and dermal fibroblast cells as our model cell lines as they all support good levels of CHIKV replication, are physiologically relevant, relatively easy to culture, express CHIKV non-structural proteins to reasonable levels and provide high-quality confocal images of CHIKV non-structural proteins.

### The cell differentiation state influences CHIKV replication levels

**Figure 3:**
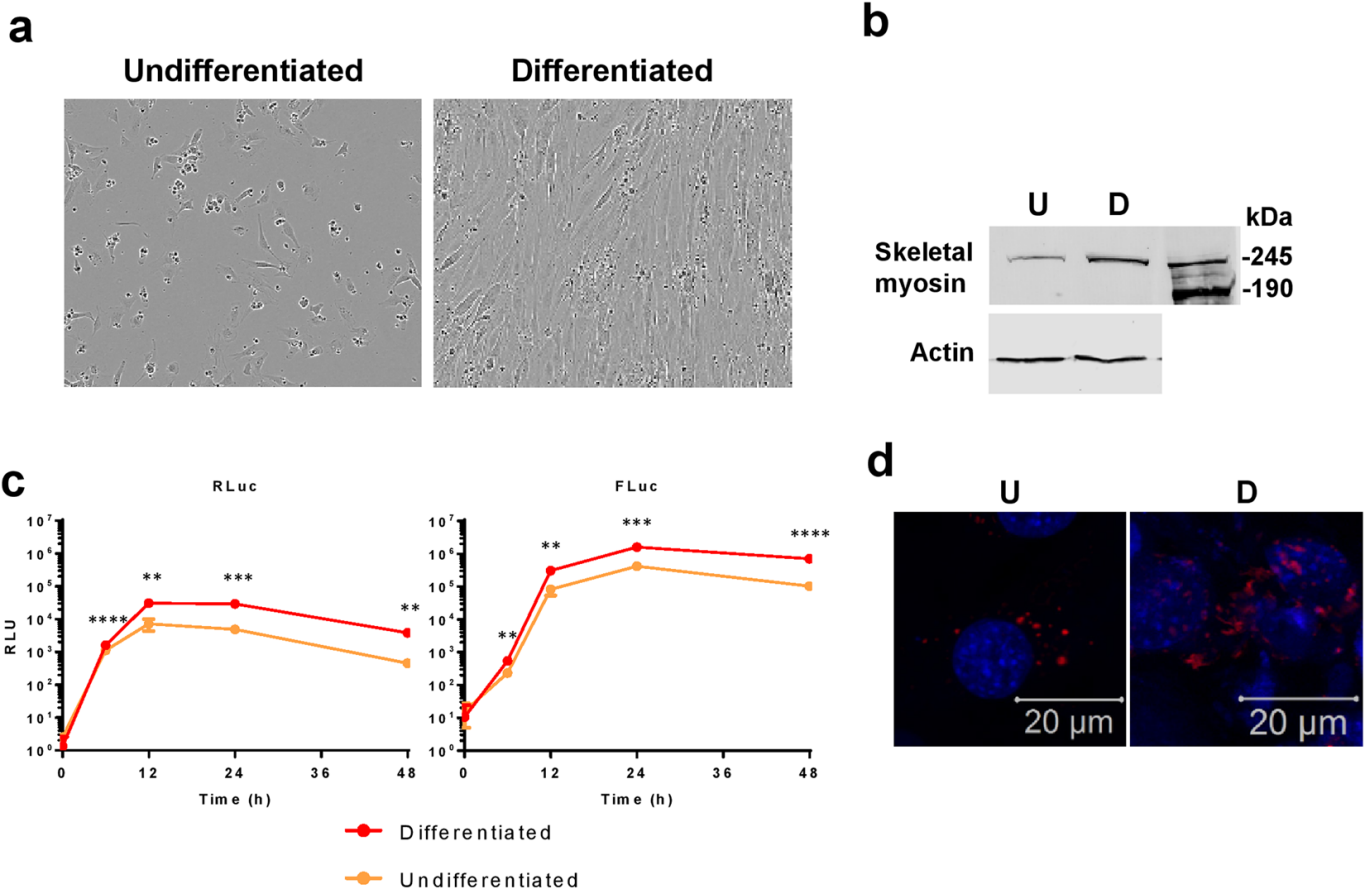
Differentiation of C2C12 cells into skeletal myotubes enhances CHIKV RNA replication. C2C12 cells were differentiated via serum starvation. **(a)** Bright field image showing the different morphology of differentiated cells compared to C2C12 cells maintained in complete culture medium (undifferentiated cells). Bright field images were obtained on an IncuCyte ZOOM instrument equipped with a 10 × objective. **(b)** Western blot for skeletal myosin, a marker of skeletal muscle tissue, is highly expressed in differentiated cells (D) compared to undifferentiated (U). **(c)** Both differentiated and undifferentiated C2C12 cells were transfected with CHIKV-D-Luc-SGR RNA. Cells were lysed at indicated time points and assayed for luciferase, data analysed by parametric T-test (**p ≤ 0.01, ***p ≤ 0.001, ****p ≤ 0.0001). **(d)** Confocal images of undifferentiated (U) and differentiated (D) cells transfected with nsP3-mCherry/SG-GLuc.

**Figure 4:**
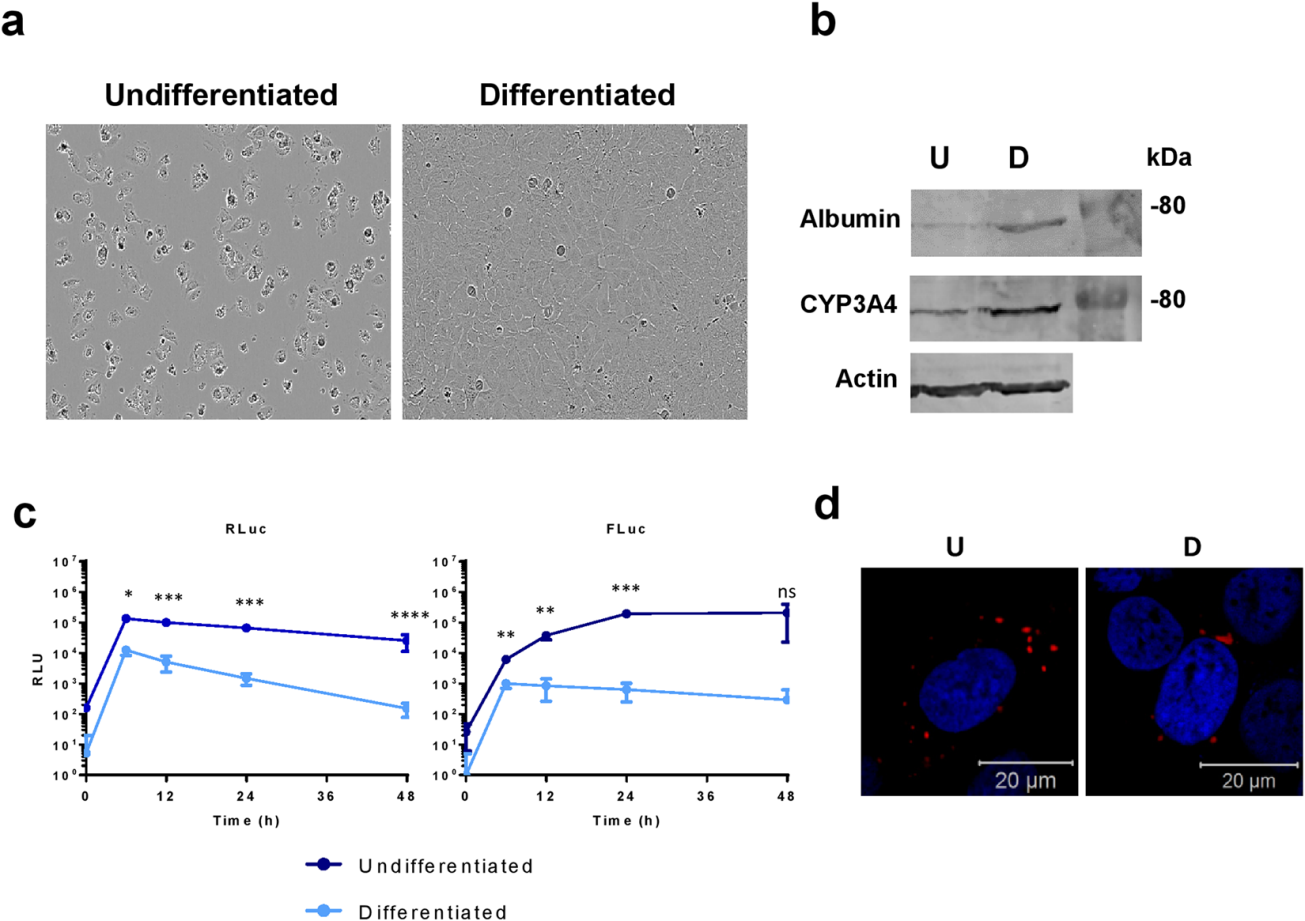
Differentiation of Huh7 cells into hepatocytes reduces CHIKV RNA replication. Huh7 cells were differentiated via addition of DMSO to complete media. **(a)** Bright field image demonstrating the morphology of differentiated cells compared to Huh7 cells maintained in complete culture medium. Bright field images were obtained on an IncuCyte ZOOM instrument equipped with a 10 × objective. **(b)** Western blot for albumin and Cytochrome P450 3A4 (CYP3A4), both are more highly expressed in differentiated cells (D) compared to undifferentiated cells (U). **(c)** Both Huh7 cells and differentiated hepatocytes were transfected with CHIKV-D-Luc-SGR RNA, cells were lysed at indicated time points and luciferase assayed, data analysed by parametric T-test. (*p ≤ 0.05, **p ≤ 0.01, ***p ≤ 0.001, ****p ≤ 0.0001). **(d)** Confocal images of undifferentiated (U) and differentiated (D) cells transfected with nsP3-mCherry/SG-GLuc replicon.

It is possible to differentiate both Huh7 and C2C12 cells into more physiologically representative states. For C2C12, reducing the concentration of serum in media (to 2% rather than 20% FCS) induces the myoblasts to differentiate to form myotubes – resembling muscle tissue *in vivo* ^30,31^ (Fig. 3a) including the upregulation of skeletal myosin (Fig. 3b). For Huh7, culturing in the presence of DMSO prevents cell division, cells enter a G_0_ state and up-regulate expression of liver-specific genes such as albumin and cytochrome P450s^32^ (Fig. 4a,b). To assess whether differentiated cells, which are more representative of their corresponding cell type *in vivo*, support higher replicative levels, cells were differentiated for 7 days and transfected with the CHIKV-D-Luc-SGR alongside non-differentiated cells plated 16 h prior.

The differentiated C2C12 cells, which resemble muscle tissue *in vivo*, supported approximately 7-fold higher levels of CHIKV SGR replication compared to undifferentiated cells at the 48 h time point (Fig. 3c). The differentiated cells also had high nsP3 expression with fewer defined puncta, more diffuse, large aggregates of nsP3 (Fig. 3d). In contrast to the differentiated C2C12 cells, the differentiated Huh7 cells exhibited dramatically reduced levels of replication compared to the undifferentiated cells, approximately 3-log fold at both 24 and 48 hpt (Fig. 4c). There was also reduced translation in the differentiated cells and the cytoplasmic organisation appeared more perinuclear, with fewer, but larger puncta (Fig. 4d).

### CHIKV infection of mammalian cells

**Figure 5:**
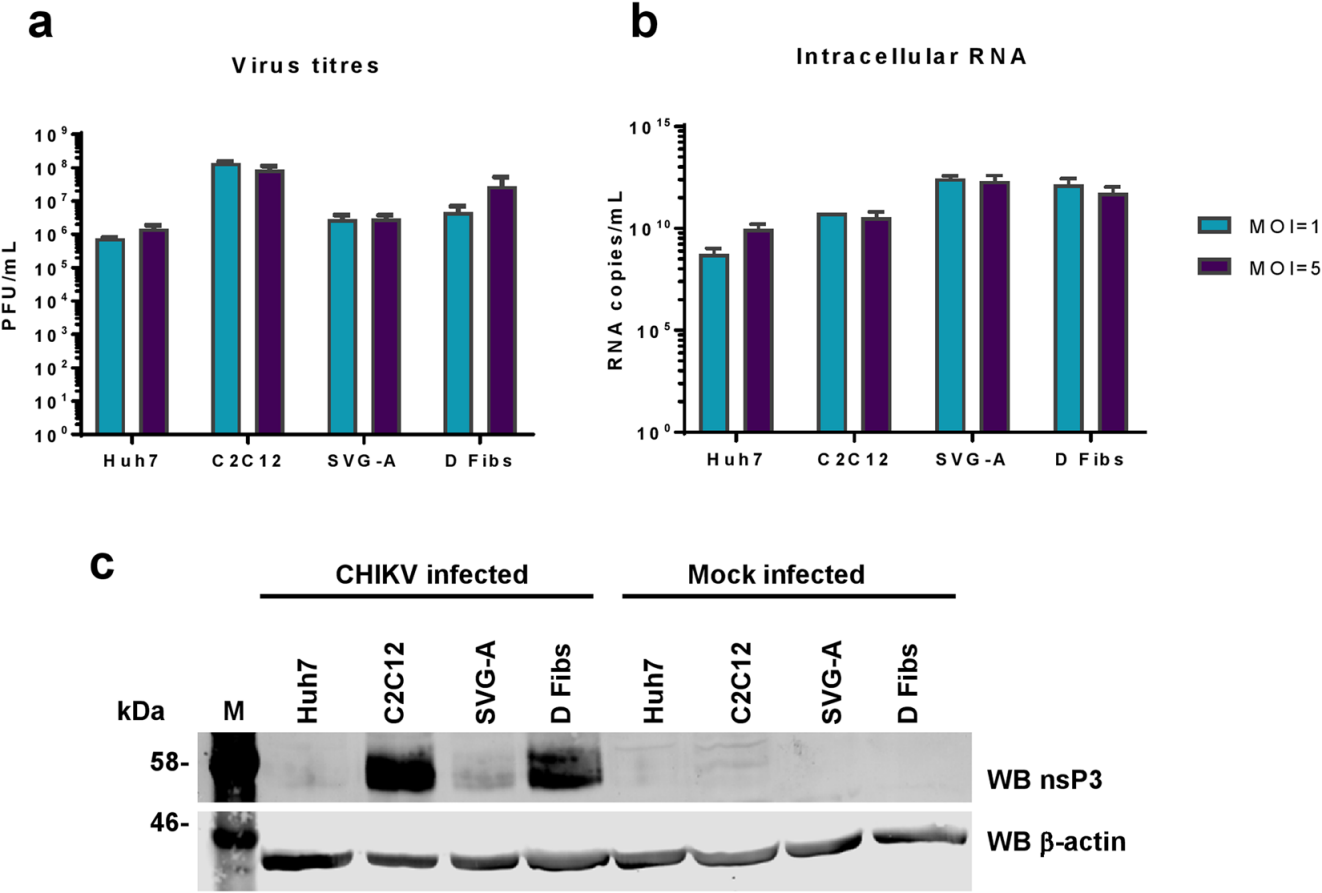
CHIKV infection of mammalian cell lines. **(a)** Virus titres. Huh7, C2C12, SVG-A, and dermal fibroblast (D Fibs) cells were infected at MOIs of either 1 or 5. At 24 hpi, the media was collected and virus titred by plaque assay (n = 3, experimental replicates). **(b)** Corresponding RNA quantification of the infected cells. Intracellular RNA was extracted using TRIzol and quantified by qRT-PCR using primers specific for nsP3 (n = 3). Values in **(a)** and **(b)** were derived from the same number of cells and are thus directly comparable. **(c)** Western blots for nsP3 in infected cells. Cells were infected with wildtype CHIKV at an MOI = 1. Cells were lysed at 24 hpi and western blot conducted for nsP3.

To confirm the findings of the SGR study, we proceeded to use infectious CHIKV. We used the corresponding ICRES clone of the ECSA strain of CHIKV: virus was produced by electroporating BHK-21 cells with *in vitro* transcribed ICRES RNA, media was collected at 24 hpt, clarified by centrifugation and titred by plaque assay, again in BHK-21 cells. Selected cell lines were then infected at an MOI of either 1 or 5, extracellular virus harvested at 24 hpi and titred by plaque assay (Fig. 5a). The relative titres produced were reflective of the replication levels observed using SGRs. C2C12 cells produced the highest titre of CHIKV, followed by dermal fibroblasts. Huh7 and SVG-A cells produced similar virus titres. This was further confirmed by qRT-PCR (Fig. 5b) and western blot analysis (Fig. 5c), where levels of genomic RNA and of nsP3 expression correlated well with the virus titres. One exception was in SVG-A cells which showed the highest levels of RNA replication, yet lower levels of both nsP3 expression and virus production compared to C2C12 and dermal fibroblasts. This suggests that these cells efficiently support CHIKV RNA replication, but do not support concomitant levels of viral protein expression and infectious virus production.

**Figure 6:**
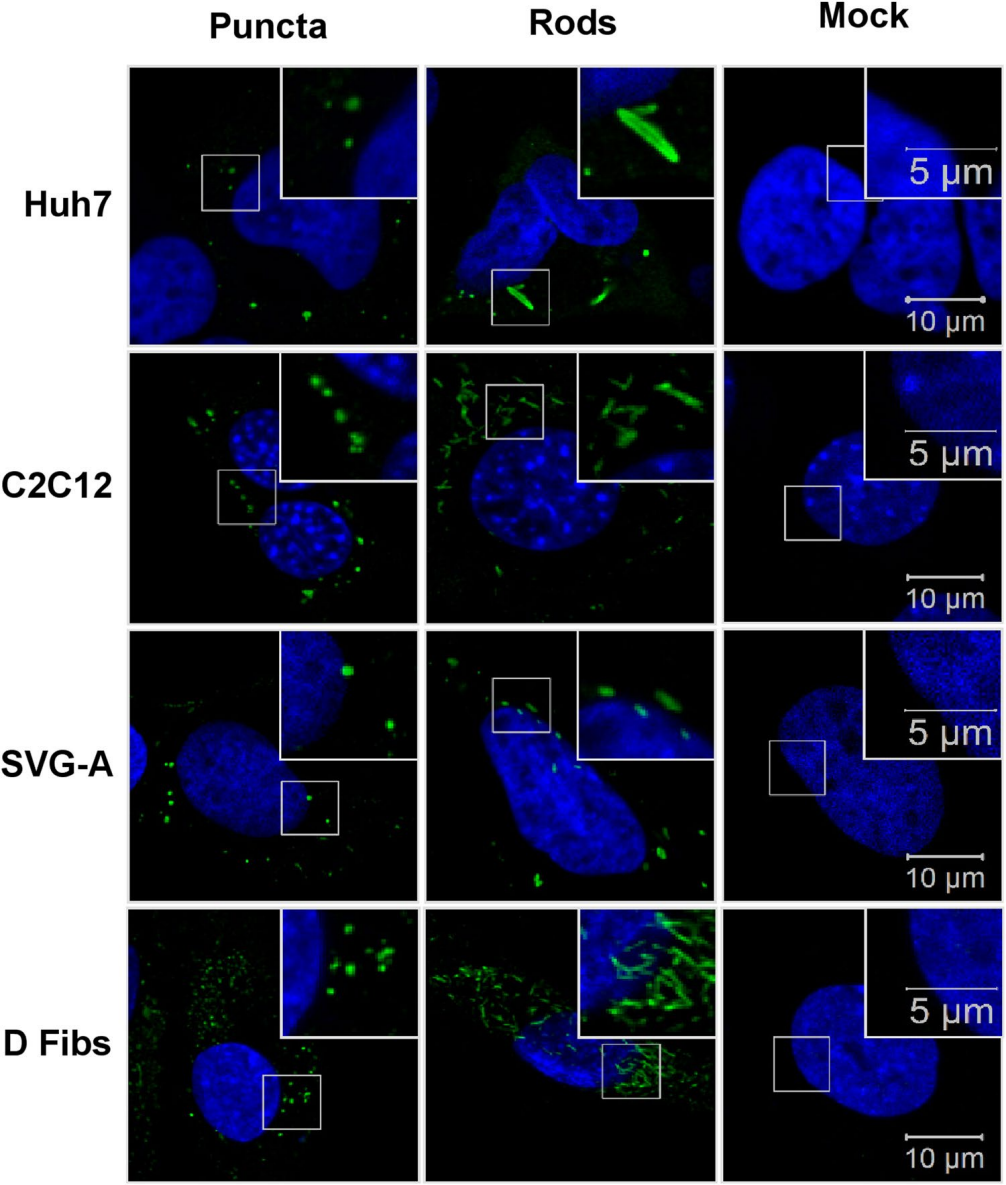
IF of nsP3 in CHIKV infected cells. Mammalian cell lines Huh7, C2C12, SVG-A, and dermal fibroblasts (D Fibs) were infected with ICRES CHIKV, fixed at 24 hpi and stained for nsP3 (green). Two different structures of nsP3 were observed; puncta, and rods in all cell lines.

We also analysed the distribution of nsP3 in infected cells by immunofluorescence (Fig. 6). Infected cells exhibited a similar distribution of nsP3 to that seen in SGR transfected cells. Overall for all cell lines, more cells contained rods rather than puncta. The levels of nsP3 expression in infected cells appeared much higher than those in the SGR-transfected cells.

### Evaluation of mosquito cell lines for replication of the CHIKV subgenomic replicon (SGR)

**Figure 7:**
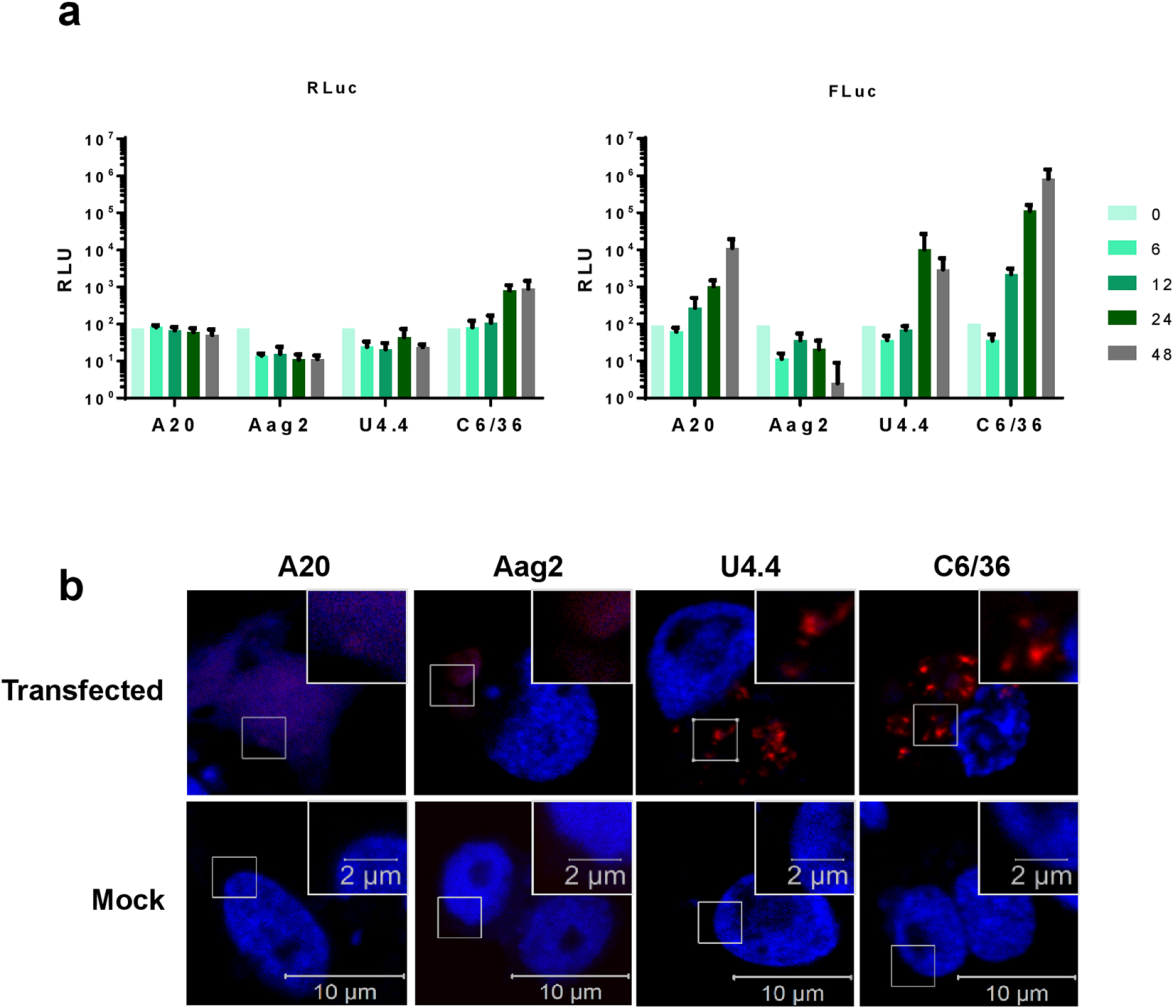
Mosquito cell lines transfected with CHIKV-D-Luc-SGR. **(a)** Aag2 and A20 cells (*Aedes aegypti)*, or C6/36 and U4.4 cells (*Aedes albopictus)*, were transfected with CHIKV-D-Luc-SGR RNA. Cell lysates were harvested at indicated time points and assayed for R-luc and F-luc as described in the legend to Fig. 1 (n = 3, experimental replicates). **(b)** Mosquito cell lines were transfected with nsP3-mCherry/SG-GLuc replicon RNA. The four lines produced very different organisations of nsP3. Each image shown is representative of nsP3 distribution observed for each cell line.

As CHIKV is an arbovirus, it was important to also study the virus lifecycle in mosquito cells. We used four mosquito cell lines as shown in Table 1. Aag2 and A20 cells are derived from embryonic *Aedes aegypti*, the mosquito responsible for CHIKV, Dengue and Zika infections. C6/36 and U4.4 cells are from *Aedes albopictus*, a mosquito more tolerant to milder climates, which has more recently become capable of infection with CHIKV of the ECSA genotype. All cell lines were transfected with the CHIKV-D-Luc-SGR and levels of nsP3 translation and replication were assayed (Fig. 7a). C6/36 cells demonstrated the highest levels of replication. Both U4.4 and A20 cells had moderate levels of replication. Aag2 cells had replicative levels below the detectable range.

We encountered many issues using CHIKV-specific antisera with mosquito cells – particularly for western blots where many non-specific bands were always observed. Therefore, to determine the distribution of nsP3 in transfected cells, cells were transfected with an SGR containing mCherry-tagged nsP3 (Fig. 7b), U4.4 and C6/36 cells exhibited organisation of nsP3 into puncta. Very few Aag2 cells were found to be nsP3 positive, indicating that low (undetectable) replication was likely due to very low transfection efficiency. Those that were positive had a diffuse appearance with no apparent organisation of nsP3. Oddly, the only A20 cells found to be nsP3 positive lacked intact nuclei, with DAPI staining throughout the cytoplasm potentially indicating that these cells are dead or dying. From this data, we concluded that the U4.4 and C6/36 cells would be good candidates to study CHIKV replication in mosquito cells. Both supported high levels of replication and detectable nsP3 expression.

### Acute infection of mosquito cells with CHIKV

**Figure 8:**
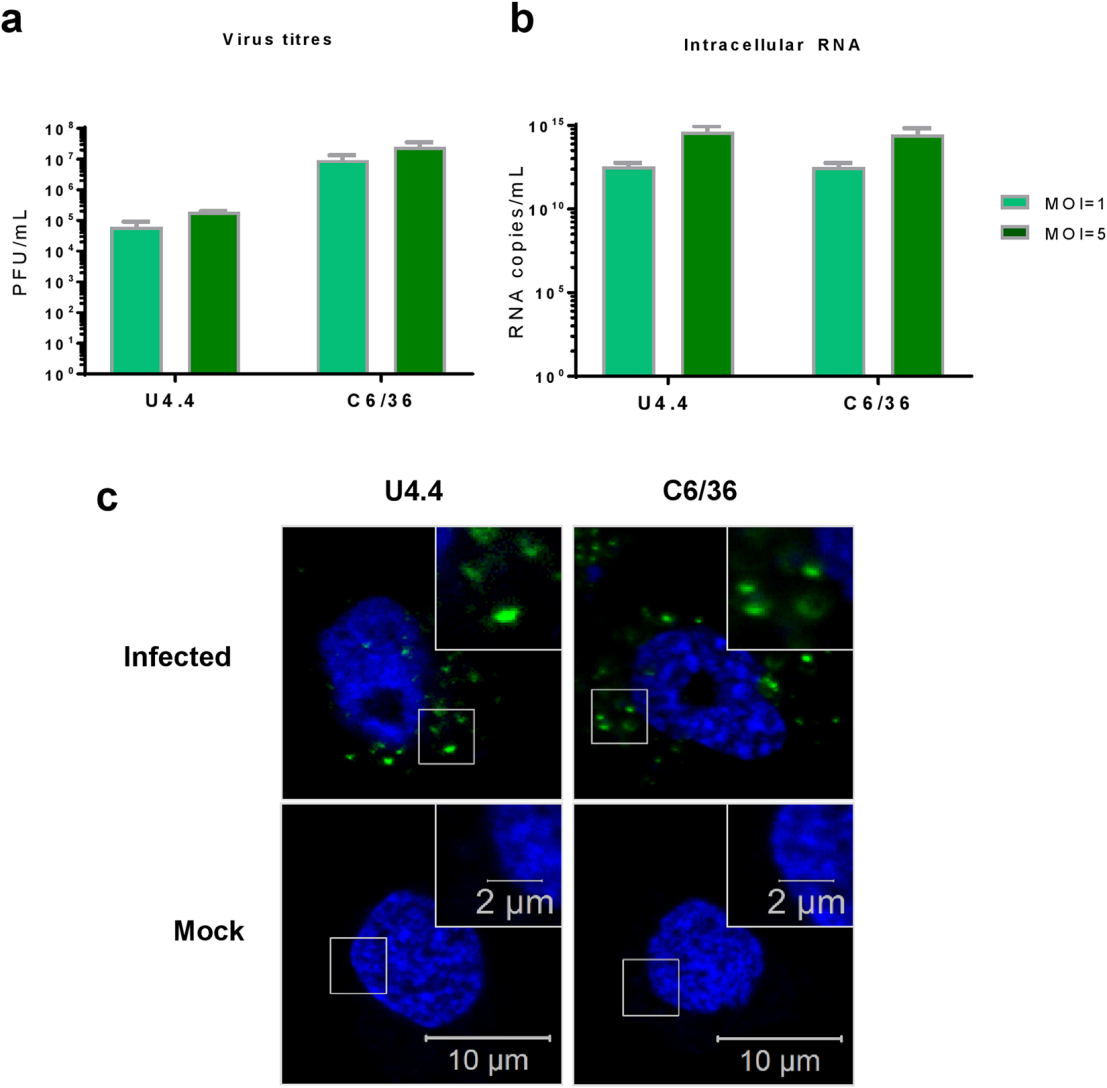
CHIKV infection of mosquito cell lines. **(a)** Virus titres. U4.4 and C6/36 cells were infected at an MOI of 1 and 5. At 24 hpi, the media was collected and virus titred by plaque assay (n = 3, experimental replicates) using BHK cells. **(b)** Corresponding RNA quantification of the infected cells. Intracellular RNA was extracted using TRIzol and quantified by qRT-PCR using primers specific for nsP3 (n = 3). Values in **(a)** and **(b)** were derived from the same number of cells and are thus directly comparable. **(c)** IF of mosquito cell lines infected with nsP3-ZsGreen virus. Both cell lines demonstrated a distinct puncta organisation of nsP3. Representative images shown.

U4.4 and C6/36 cells were infected with CHIKV similarly to mammalian cells. Resulting titres are shown in Fig. 8a and the corresponding CHIKV RNA levels are shown by qRT-PCR (Fig. 8b). Similarly to SGR assays, C6/36 cells produced higher levels of virus than U4.4 cells, though the intracellular RNA levels of the two cell lines were very similar. Both cell lines produced moderately high titres of virus which correlated well with the SGR luciferase data.

To image nsP3 in infected cells, we used a CHIKV virus construct containing ZsGreen tagged nsP3 instead of antibody staining^28^. Cells were infected with this virus for 24 h, fixed and DAPI stained prior to imaging (Fig. 8c). For both mosquito cell lines, only puncta were observed, though they varied in size. Smaller puncta were present in U4.4 cells whereas C6/36 cells exhibited a wider range of puncta size.

## Discussion

CHIKV has been shown to be able to infect and replicate in a wide range of cell lines, many of which are regularly used for CHIKV research, but most are physiologically irrelevant such as BHK-21, HeLa and Vero cells. Here, we have established a set of ‘model’ cell lines, both mammalian and mosquito, which are physiologically relevant and useful for CHIKV research.

We initially transfected ten mammalian cell lines with a dual luciferase CHIKV SGR RNA. From the physiologically relevant cell lines (i.e. liver/muscle/brain/fibroblast cells), Huh7, C2C12, RD, SVG-A, and dermal fibroblasts cells supported moderate to high levels of replication and all exhibited both puncta and rod organisation of nsP3. Vero E6 and HeLa, commonly used in CHIKV studies, had reduced replication at the later time points. The IF data indicates this was due to a high level of cell death in transfected cells as most nsP3 positive cells showed nuclear blebbing. In this study, A549 cells exhibited limited levels of replication that reduced over time. This is in agreement with the literature which has shown that CHIKV can bind and enter A549 cells but replication is somehow impeded^33–35^. In our study, translation of the non-structural proteins did occur, but RNA replication was restricted. The immunofluorescence data shows a unique nsP3 organisation in the majority of transfected A549 cells as they contained much smaller but more numerous puncta, possibly indicating a spatial restriction of the CHIKV non-structural proteins within cells. Overall, there was no obvious correlation between levels of RNA replication and nsP3 organisation seen in the IF experiments. However, in the virus-infected cells, the cell lines that produced the highest titres, C2C12 and dermal fibroblasts, appear to have more cells exhibiting the rod-like organisation. Thus, the rod-organisation may allow for more efficient replication, or could indicate a later stage in the virus lifecycle. More research is required to determine the significance of the rod and puncta organisations of nsP3, as discussed previously^29^. Most cell lines produced detectable levels of wildtype nsP3, as shown by western blot. Predictably, the two cell lines with the highest levels of replication; C2C12 and BHK-21, exhibited the highest nsP3 expression.

Taken together, we concluded that the most physiologically relevant and tractable cell lines for all practical applications tested, were Huh7 (hepatocytes), C2C12 (myoblasts), SVG-A (glial cells) and dermal fibroblasts. This was confirmed using infectious CHIKV, where all four cell lines produced high levels of virus, roughly correlating with the SGR data, although Huh7, SVG-A, and dermal fibroblast cells appeared to support higher levels of replication in the infectious system than the SGR. All infected cell lines exhibited comparable IF and western blot data. Interestingly, when analysed by IF, cell lines infected with CHIKV exhibited both puncta and rods of nsP3. However, more cells contained rods, and individual cells appeared to contain higher quantities of nsP3. These differences in replication and nsP3 organisation from SGR and virus highlight the importance of treating replicon-based data with caution. Although SGRs are the most appropriate system to study virus replication under BSL2 conditions, they do not fully recapitulate the infectious virus lifecycle.

Both C2C12 and Huh7 cell lines can be differentiated via different methods making them more representative of their tissue types *in vivo*. C2C12 cells fuse into large, multinucleated cells and upregulate skeletal-muscle protein expression such as skeletal myosin, resembling muscle tissue *in vivo* ^30,31^. In contrast, for Huh7 cells differentiation manifests as growth arrest and an up-regulation of liver-specific proteins such as albumin and cytochrome p450^32^. On a multicellular level, they form large ‘rafts’ of differentiated cells, resembling hepatocyte plates in the liver. For differentiated muscle, CHIKV SGR replication was enhanced approximately seven-fold over C2C12 cells. We observed high levels of nsP3 expression in differentiated cells – much more so than that seen in undifferentiated C2C12 cells. This may be due to the presence of muscle tissue-specific factors that are enhancing replication. The organisation of nsP3 was also less punctate and more diffuse, potentially due to the nature of multinucleated, fused cells which would have a different subcellular organisation when compared to single, undifferentiated cells. For instance, skeletal muscle tissue is known to contain higher quantities of mitochondria^36^. Interestingly, differentiated liver cells exhibited much lower levels of replication compared to Huh7 cells. Translation of the non-structural proteins was reduced in differentiated cells and the distribution of nsP3 in was slightly altered. In differentiated cells, nsP3 was found in much larger puncta that were less numerous and were only found in the perinuclear region. The reduced replication and restricted translation could potentially be due to the differentiated cells being in growth arrest. However, this reduced replication does not necessarily indicate that these differentiated cells are a less representative model of CHIKV replication in the liver as most liver cells *in vivo* will not be dividing.

As CHIKV is an arbovirus, it was important to study the virus in its mosquito vector as well as the mammalian host. We tested four mosquito cell lines for their use in CHIKV research. From the SGR luciferase assay, all were able to support RNA replication apart from Aag2 cells. This may be due to poor transfection efficiency as reported by various laboratories. It may also be due to the persistent infection of Aag2 cells with the cell fusing agent virus^37,38^ (CFAV). CFAV is an insect-cell specific flavivirus that induces infected cells to undergo syncytium formation, where cells fuse to form enlarged, multi-nucleated cells, induced by the fusion of the virus with the cell membrane^39^. This remodelling, or the presence of an established viral infection, may have prevented SGR replication in these cells. C6/36 cells demonstrated the highest levels of CHIKV SGR replication, which was expected as these cells have a frame-shift mutation in the *Dcr2* gene leading to expression of a truncated and inactive Dicer2 which renders the anti-viral RNAi system inactive^40,41^. From the microscopy images, both *Aedes albopictus* cell lines, U4.4 and C6/36, exhibited very bright nsP3 puncta, with many cells being nsP3 positive. In contrast, very few Aag2 cells expressed nsP3 and those that did had a diffuse appearance, with no defined puncta. This apparent lack of organisation and the number of transfected cells may explain the lack of RNA replication. Although, in the case of the A20 cells, which did support SGR replication, the only nsP3 positive cells observed had no defined nuclei with DAPI staining throughout the cytoplasm. This observation is suggestive of high cytotoxicity, though it is unclear whether this is caused by the presence of CHIKV proteins or the transfection reagent. Therefore, we decided to continue with U4.4 and C6/36 cell lines for virus infection. Both lines produced high virus titres at both MOIs of 1 and 5 and exhibited similar nsP3 organisation to SGR transfected cells. These two lines also provide an interesting comparison for future studies as they are both *Aedes albopictus* in origin but the C6/36 cells lack an intact RNAi system, allowing for further insight into cellular response to virus infection. Unfortunately, the majority of antibodies tested with mosquito cell extracts exhibited high cross-reactivity with cellular proteins making western blotting technically challenging. For this reason, we avoided using antibodies for confocal imaging, opting instead to use fluorescently-tagged protein constructs.

Here we have demonstrated the use of four physiologically relevant mammalian cell lines, and two mosquito cell lines in CHIKV *in vitro* research. We have shown their abilities to replicate both CHIKV SGR and infectious virus, and for imaging nsP3 via confocal microscopy.

## Materials and Methods

### Cell culture

All mammalian cell lines were maintained at 37 °C with 5% CO_2_. Huh7, SVG-A, Dermal Fibroblasts, HepG2, RD, BHK-21, A549, HeLa and Vero E6 cells were maintained in DMEM supplemented with 10% FCS, 0.5 mM non-essential amino acids and penicillin-streptomycin (100 units/mL). C2C12 cells were maintained in DMEM supplemented with 20% FCS and penicillin-streptomycin (100 units/mL). The dermal fibroblast cells were formed using primary dermal fibroblast cells, isolated from a healthy patient^42^ (ethics number BIOSCI09-001, provided by Dr Martin Stacey, University of Leeds) and transformed using a lentivirus expressing hTERT.

To differentiate Huh7 cells, cells were grown to sub-confluency before media changed to complete media with 2% DMSO. To differentiate C2C12 cells, the cells were grown to 80% confluency then media changed to DMEM supplemented with 2% FCS. For both cell lines, cells were maintained in differentiation media for 7 days. Media was replaced every 2 days. Bright field images were obtained on an IncuCyte ZOOM instrument equipped with a 10× objective. All insect cell lines (A20, Aag2, U4.4 and C6/36) were maintained in Leibovitz’s L-15 media supplemented with 10% FBS, 10% tryptose phosphate broth and penicillin-streptomycin (100 units/mL). Cells were incubated at 28 °C without CO_2_.

### Plasmids - SGR and Virus

The CHIKV dual luciferase SGR (CHIKV-D-Luc-SGR), CHIKV wildtype-nsP3 firefly luciferase SGR (CHIKV-FLuc-SGR), CHIKV nsP3-mCherry SGR and CHIKV ICRES virus constructs are derived from the ECSA genotype LR2006-OPY1 isolate of CHIKV. CHIKV SGR and virus SP6-plasmids are as described^28^.

### *In vitro* transcription

SGR and virus plasmids were linearised using NotI-HF, and *in vitro* transcribed using the mMessage mMachine SP6 transcription kit (Ambion). RNA was purified using the RNeasy mini kit (Qiagen), aliquoted and stored at −80 °C.

### SGR Transfections

Cells were plated in 24 well plates (1 × 10^5^ cells/well) 16 h prior to transfection. Before transfection, cells were washed with PBS and media replaced with Optimem (LifeTechnologies). For each well, 250 ng of RNA and 1 µL of Lipofectamine 2000 (Invitrogen) in 100 µL Optimem was prepared as a master mix, incubated for 20 minutes prior to addition to each well. Cells were incubated for 4 h at 37 °C for 4 h, then washed and media replaced with complete media.

### Luciferase assays

For luciferase assays, cells were transfected as above and harvested by washing cells in PBS then lysing in passive lysis buffer (PLB, Promega). Samples were stored at −20 °C, and defrosted prior to reading using the dual-luciferase reporter assay system (Promega) and FLUOStar optima microplate reader (BMG Labtech). Luciferase was normalised to mock-transfected control.

### Virus production

All virus stocks were produced in BHK-21 cells. 1 µg of virus RNA was added to the bottom of a precooled 4 mm electroporation cuvette (Cell Projects), 1.2 × 10^6^ cells in DEPC-treated PBS were added, mixed and electroporated at 260 V for 25 ms using GenePulser Xcell (Bio-Rad). Cells were then resuspended in 10 mL complete growth media and seeded in a T75 flask. Virus-containing supernatant was removed at 24 h post-electroporation, clarified by centrifugation (1000 × g, 3 min), aliquoted and stored at −80 °C. Virus concentration was calculated by plaque assay (see below).

### Virus Infection

Cells were plated 16 h prior to infection. Cells were washed with PBS, virus diluted in media was added. Plates were rocked for 5 min, incubated for 1 h at 37 °C, virus removed, cells washed and complete media replaced.

### Plaque assays

All plaque assays were performed in BHK-21 cells. Cells were seeded 16 h prior to assay in 6 well plates (4 × 10^5^ cells/well). Serial dilutions of virus stocks were made in complete media. Cells were washed with PBS and 180 µL of virus dilution added to wells. Plates were rocked for 5 min prior to incubation at 37 °C for 1 h. Virus solution was removed, cells washed in PBS and layered with 2 mL methyl cellulose (MC, 0.8%) in complete media. Cells were then further incubated for 48 h, where MC was removed, cells fixed in 10% formaldehyde for 30 min and stained crystal violet (0.5%) for 30 min. Cells were washed with water until plaques became visible.

### qRT-PCR

Total cell RNA was extracted from cells using TRIzol (ThermoFisher). All qRT-PCRs were performed using the One-step MESA green qRT-PCR MasterMix for SYBR assay kit (Eurogentec) following manufacturer’s recommended protocol. Primers used were specific to the alphavirus unique domain of nsP3 (forward: GCGCGTAAGTCCAAGGGAAT; reverse AGCATCCAGGTCTGACGGG as used by Chiam *et al*.^43^).

### Western Blots

Cells were transfected or infected as above, then harvested in 100 µL PLB. Protein concentration of samples was calculated using a Bradford assay. For nsP3 blots, cell lysate containing 30 µg of total protein in Laemmli buffer were incubated at 95 °C for 5 min prior to loading on a 10% SDS-PAGE gel. Proteins were then transferred from gels onto membranes (Immobilon FL, Merck Millipore) using a semi-dry blotter for 1 h at 15 V. Membranes were blocked using blocking buffer (LICOR) for 20 min at room temperature prior to incubation with primary antibodies; rabbit anti-nsP3^44^ or mouse anti-actin (Sigma) prepared in TBS. Membranes were incubated with primary antibody rocking overnight at 4 °C. Membranes were washed in TBS then secondary antibodies (LICOR) added for 1 h at rt. Membranes were then washed in TBS + 0.1% Tween, then dH_2_O prior to drying in the dark. Blots were imaged using the LICOR Odyssey scanner.

### Immunofluorescence

Cells grown on coverslips were transfected or infected with the nsP3-mCherry CHIKV SGR, wild type or nsP3-ZsGreen expressing viruses as described above. Cells were washed with PBS then fixed with 4% paraformaldehyde for 10 min at room temperature. For samples containing fluorescently tagged nsP3, cells were washed in PBS and dipped in dH_2_O then mounted using prolong gold containing DAPI (Life Technologies) onto microscope slides. For nsP3 staining in samples infected with wild type virus, cells were permeabilised in 0.5% Triton-X-100 for 10 min, washed in PBS, blocked in 2% BSA for 1 h, washed, nsP3 antibody applied for 1 h, washed and secondary (donkey-anti rabbit 488, Life Technologies) applied for 1 h, washed, dipped in dH_2_O then mounted in prolong gold with DAPI. Cells were imaged using the Zeiss LSM 700 confocal microscope at 40x magnification for mammalian cells and 63x magnification for mosquito cells. Images were processed using Zen black software.

### Data availability

The datasets generated during and/or analysed during the current study are available from the corresponding author on reasonable request.

## Electronic supplementary material

Supplementary Figure S1

## References

[1] Schwartz O, Albert ML (2010). Biology and pathogenesis of chikungunya virus. Nat Rev Micro.

[2] Mavale M (2010). Venereal Transmission of Chikungunya Virus by Aedes aegypti Mosquitoes (Diptera: Culicidae). Am. J. Trop. Med. Hyg..

[3] Weaver SC, Forrester NL (2015). Chikungunya: Evolutionary history and recent epidemic spread. Antiviral Res..

[4] Ng LC, Hapuarachchi HC (2010). Tracing the path of Chikungunya virus—Evolution and adaptation. Infect. Genet. Evol..

[5] Brady OJ (2013). Modelling adult Aedes aegypti and Aedes albopictus survival at different temperatures in laboratory and field settings. Parasit. Vectors.

[6] Lanciotti RS, Valadere AM (2014). Transcontinental movement of Asian Genotype Chikungunya virus. Emerg. Infect. Dis..

[7] Josseran L (2006). Chikungunya Disease Outbreak, Reunion Island. Emerg. Infect. Dis..

[8] Mattar S (2015). Outbreak of Chikungunya virus in the north Caribbean area of Colombia: clinical presentation and phylogenetic analysis. J. Infect. Dev. Ctries..

[9] Feldstein LR, Ellis EM, Rowhani-Rahbar A, Halloran EM, Ellis BR (2016). The First Reported Outbreak of Chikungunya in the U.S. Virgin Islands, 2014-2015. Am. J. Trop. Med. Hyg..

[10] Delisle, E. *et al*. Chikungunya outbreak in Montpellier, France, September to October 2014. *Euro Surveill*. *Bull*. *Eur*. *Sur Mal*. *Transm*. *Eur*. *Commun*. *Dis*. *Bull*. **20**, (2015). 10.2807/1560-7917.es2015.20.17.21108 25955774

[11] Liumbruno GM (2008). The Chikungunya epidemic in Italy and its repercussion on the blood system. Blood Transfus..

[12] Paupy C, Delatte H, Bagny L, Corbel V, Fontenille D (2009). Aedes albopictus, an arbovirus vector: From the darkness to the light. Forum Chikungunya.

[13] Kraemer MU (2015). The global distribution of the arbovirus vectors Aedes aegypti and Ae. albopictus. eLife.

[14] Weaver SC, Reisen WK (2010). Present and future arboviral threats. Antiviral Res..

[15] Lo Presti A, Lai A, Cella E, Zehender G, Ciccozzi M (2014). Chikungunya virus, epidemiology, clinics and phylogenesis: A review. Asian Pac. J. Trop. Med..

[16] Hoarau, J. J. & Jaffar Bandjee, M. C. Persistent chronic inflammation and infection by Chikungunya arthritogenic alphavirus in spite of a robust host immune response. *J Immunol* **184** (2010). 10.4049/jimmunol.0900255 20404278

[17] Gunn BM (2012). Mannose Binding Lectin Is Required for Alphavirus-Induced Arthritis/Myositis. PLoS Pathog.

[18] Gérardin P (2008). Multidisciplinary Prospective Study of Mother-to-Child Chikungunya Virus Infections on the Island of La Réunion. PLoS Med.

[19] Rampal SM, Meena H (2007). Neurological complications in Chikungunya fever. J. Assoc. Physicians India.

[20] Weaver SC, Osorio JE, Livengood JA, Chen R, Stinchcomb DT (2012). Chikungunya virus and prospects for a vaccine. Expert Rev. Vaccines.

[21] Deeba F (2016). Chikungunya virus: recent advances in epidemiology, host pathogen interaction and vaccine strategies. Pathog. Dis..

[22] Broeckel R, Haese N, Messaoudi I, Streblow DN (2015). Nonhuman Primate Models of Chikungunya Virus Infection and Disease (CHIKV NHP Model). Pathogens.

[23] Caglioti C (2013). Chikungunya virus infection: an overview. New Microbiol..

[24] Gallegos KM, Drusano GL, D’Argenio DZ, Brown AN (2016). Chikungunya Virus: *In Vitro* Response to Combination Therapy with Ribavirin and Interferon-α2a. J. Infect. Dis..

[25] Zandi, K. A Real-Time Cell Analyzing Assay for Identification of Novel Antiviral Compounds against Chikungunya Virus. in *Chikungunya Virus: Methods and Protocols* (eds Chu, H. J. J. & Ang, K. S.) 255–262 (Springer New York, 2016). 10.1007/978-1-4939-3618-2_23 27233278

[26] Reid SP (2015). Sphingosine kinase 2 is a chikungunya virus host factor co-localized with the viral replication complex. Emerg. Microbes Infect..

[27] von Rhein C (2016). Curcumin and Boswellia serrata gum resin extract inhibit chikungunya and vesicular stomatitis virus infections *in vitro*. Antiviral Res..

[28] Pohjala L (2011). Inhibitors of Alphavirus Entry and Replication Identified with a Stable Chikungunya Replicon Cell Line and Virus-Based Assays. PLOS ONE.

[29] Remenyi R, Roberts GC, Zothner C, Merits A, Harris M (2017). SNAP-tagged Chikungunya Virus Replicons Improve Visualisation of Non-Structural Protein 3 by Fluorescence Microscopy. Sci. Rep..

[30] Lawson MA, Purslow PP (2000). Differentiation of Myoblasts in Serum-Free Media: Effects of Modified Media Are Cell Line-Specific. Cells Tissues Organs.

[31] Burattini S (2004). C2C12 murine myoblasts as a model of skeletal muscle development: morpho-functional characterization. Eur. J. Histochem. EJH.

[32] Choi S, Sainz B, Corcoran P, Uprichard S, Jeong H (2009). Characterization of increased drug metabolism activity in dimethyl sulfoxide (DMSO)-treated Huh7 hepatoma cells. Xenobiotica Fate Foreign Compd. Biol. Syst..

[33] Sourisseau M (2007). Characterization of Reemerging Chikungunya Virus. PLoS Pathog.

[34] Salvador B, Zhou Y, Michault A, Muench MO, Simmons G (2009). Characterization of Chikungunya pseudotyped viruses: Identification of refractory cell lines and demonstration of cellular tropism differences mediated by mutations in E1 glycoprotein. Virology.

[35] Olagnier D (2014). Inhibition of Dengue and Chikungunya Virus Infections by RIG-I-Mediated Type I Interferon-Independent Stimulation of the Innate Antiviral Response. J. Virol..

[36] Porter C, Wall BT (2012). Skeletal muscle mitochondrial function: is it quality or quantity that makes the difference in insulin resistance?. J. Physiol..

[37] Scott JC (2010). Comparison of Dengue Virus Type 2-Specific Small RNAs from RNA Interference-Competent and –Incompetent Mosquito Cells. PLoS Negl. Trop. Dis..

[38] Zhang G, Etebari K, Asgari S (2016). Wolbachia suppresses cell fusing agent virus in mosquito cells. J. Gen. Virol..

[39] Cook, S. *et al*. Isolation of a new strain of the flavivirus cell fusing agent virus in a natural mosquito population from Puerto Rico. *J*. *Gen*. *Virol*. **87**, 735–748. 10.1099/vir.0.81475-0 16528021

[40] Brackney DE (2010). C6/36 Aedes albopictus Cells Have a Dysfunctional Antiviral RNA Interference Response. PLoS Negl Trop Dis.

[41] Morazzani EM, Wiley MR, Murreddu MG, Adelman ZN, Myles KM (2012). Production of Virus-Derived Ping-Pong-Dependent piRNA-like Small RNAs in the Mosquito Soma. PLOS Pathog..

[42] Macleod T (2016). Neutrophil Elastase-mediated proteolysis activates the anti-inflammatory cytokine IL-36 Receptor antagonist. Sci. Rep..

[43] Chiam CW (2013). Real-time polymerase chain reaction for diagnosis and quantitation of negative strand of chikungunya virus. Diagn. Microbiol. Infect. Dis..

[44] Varjak M, Žusinaite E, Merits A (2010). Novel Functions of the Alphavirus Nonstructural Protein nsP3 C-Terminal Region. J. Virol..

[45] Ross-Thriepland D, Harris M (2014). Insights into the Complexity and Functionality of Hepatitis C Virus NS5A Phosphorylation. J. Virol..

[46] Parker F, White K, Phillips S, Peckham M (2016). Promoting differentiation of cultured myoblasts using biomimetic surfaces that present alpha-laminin-2 peptides. Cytotechnology.

[47] Gee GV, Manley K, Atwood WJ (2003). Derivation of a JC virus-resistant human glial cell line: implications for the identification of host cell factors that determine viral tropism. Virology.

[48] Willberg CB (2007). Protection of Hepatocytes from Cytotoxic T Cell Mediated Killing by Interferon-Alpha. PLOS ONE.

[49] De Colibus L (2014). More-powerful virus inhibitors from structure-based analysis of HEV71 capsid-binding molecules. Nat Struct Mol Biol.

[50] Dove BK (2012). A quantitative proteomic analysis of lung epithelial (A549) cells infected with 2009 pandemic influenza A virus using stable isotope labelling with amino acids in cell culture. PROTEOMICS.

[51] Cesur Ö (2015). The Subcellular Localisation of the Human Papillomavirus (HPV) 16 E7 Protein in Cervical Cancer Cells and Its Perturbation by RNA Aptamers. Viruses.

[52] Hover S (2016). Modulation of Potassium Channels Inhibits Bunyavirus Infection. J. Biol. Chem..

[53] Pudney M, Varma MGR, Leake CJ (1979). Establishment of cell lines from larvae of culicine (Aedes species) and anopheline mosquitoes. TCA Man. Tissue Cult. Assoc..

[54] Siu RWC (2011). Antiviral RNA Interference Responses Induced by Semliki Forest Virus Infection of Mosquito Cells: Characterization, Origin, and Frequency-Dependent Functions of Virus-Derived Small Interfering RNAs. J. Virol..

