# Supplementary material for: Evaluation of a range of mammalian and mosquito cell lines for use in Chikungunya virus research
Source: Sci Rep. 2017 Nov 7;7:14641. doi: 10.1038/s41598-017-15269-w (PMC5677012; doi:10.1038/s41598-017-15269-w)
Supplement: Supplementary file 1 — Supplementary Figure S1 [file 41598_2017_15269_MOESM1_ESM.pdf]

# Evaluation of a range of mammalian and mosquito cell lines for use in Chikungunya virus research.

Grace Roberts, Carsten Zothner, Roland Remenyi, Andres Merits, Nicola J. Stonehouse and Mark Harris

Supplementary Information

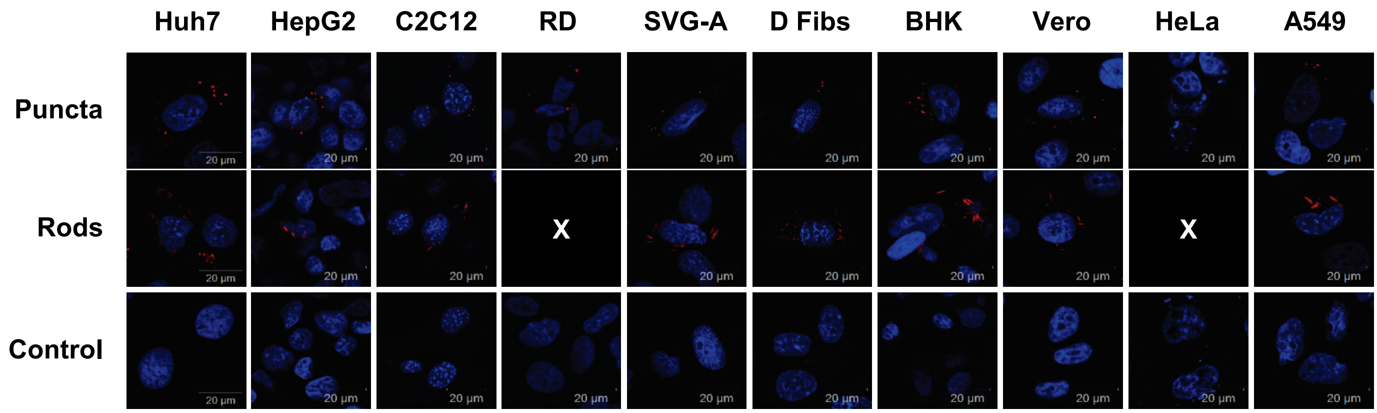


**Figure S1. Localisation of mCherry-tagged-nsP3 in 10 mammalian cell lines.** Mammalian cell lines were transfected with nsP3-mCherry/SG-Gluc replicon RNA using Lipofectamine 2000. Two defined species of nsP3 structures were observed: puncta and rods. Spaces with an ”x” indicate that that particular nsP3 structure was not observed for that cell line.

# Fig S2: Full length western blot images

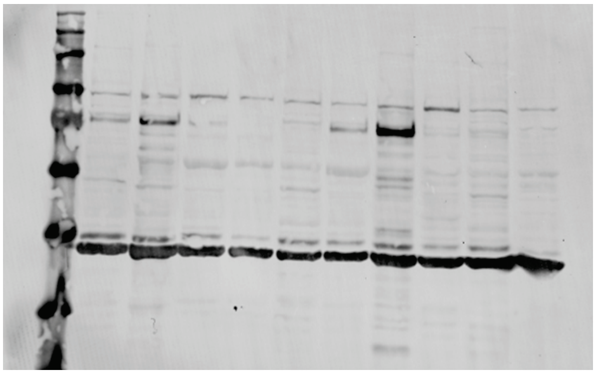

Fig 1d

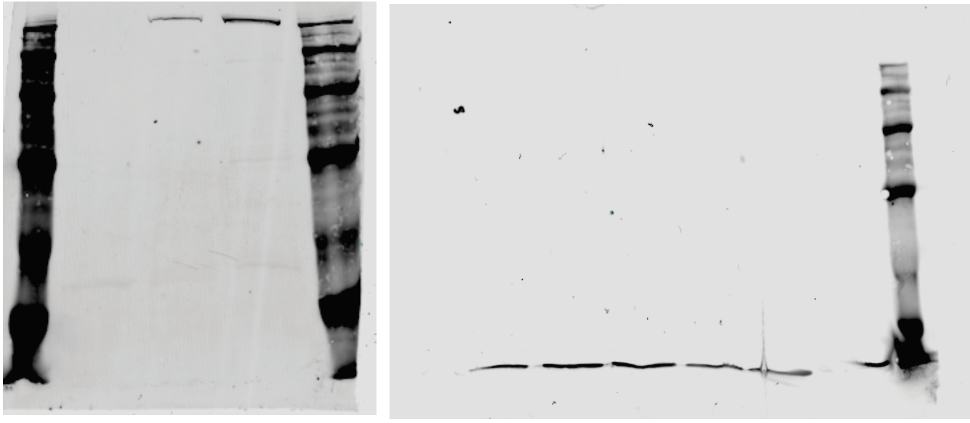

Fig 3b

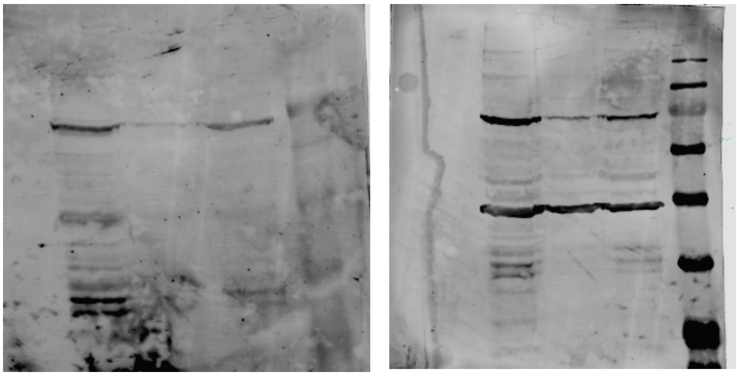

Fig 4b

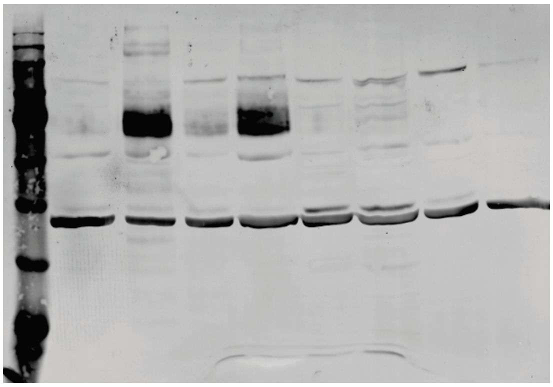

Fig 5b
